# Statistics of Rupture of the Urinary Bladder

**Published:** 1851-06

**Authors:** 


*A contribution to the statistics of Rupture of the Urinary Bladder; with a table of seventy-eight eases.*—By Stephen Smith, M. D., Assistant surgeon to Bellevue Hospital, New-York: John F. Trow, printer, 49 Ann-street, 1851.

This is one of the most valuable contributions to surgical, pathological, and medico-legal science, of the year, and will rank its author among the most laborious and accurate staticians of our country. F. H. H.

